# Journeying through Dementia, a community-based self-management intervention for people aged 65 years and over: a feasibility study to inform a future trial

**DOI:** 10.1186/s40814-015-0039-6

**Published:** 2015-11-30

**Authors:** Kirsty Sprange, Gail A. Mountain, Katy Shortland, Claire Craig, Daniel Blackburn, Peter Bowie, Kirsty Harkness, Maggie Spencer

**Affiliations:** 1 Nottingham Clinical Trials Unit, University of Nottingham, Queens Medical Centre, Nottingham, NG7 2UH UK; 2 School of Health and Related Research (ScHARR), Regent Court, University of Sheffield, Regent Street, Sheffield, S1 4DA UK; 3 National Institute for Health Research, Sheffield Clinical Research Facility, Royal Hallamshire Hospital, Sheffield, S10 2JF UK; 4 Centre for Health & Social Care Research, Collegiate Campus, Hallam University, Montgomery House, Sheffield, S10 2BP UK; 5 Sheffield Institute for Translational Neuroscience (SITraN), University of Sheffield, Sheffield, S10 2HQ UK; 6 Sheffield Health and Social Care Trust, Fulwood House, Old Fulwood Road, Sheffield, S10 3TH UK; 7 Royal Hallamshire Hospital, Glossop Road, Sheffield, S10 2JF UK

**Keywords:** Dementia, Self-management, Quality of life, Community, Manualised intervention

## Abstract

**Background:**

A study to determine the feasibility of conducting a future population-based trial into a self-management intervention for community-living adults with early stage dementia included evaluation of intervention content and modes of delivery, staffing requirements, recruitment methods and the utility and usability of patient reported outcomes.

**Methods:**

Participants identified through memory clinics in one city took part in an intervention called ‘Journeying through Dementia’. The 12-week programme incorporating four individual sessions with one of the facilitators encourages participants to engage in discussion and activities related to health and well-being positioning them as the expert enabling long-term behavioural change. Participants (*n* = 10) and their nominated carers (*n* = 7) were all asked to complete selected outcomes at baseline, 8 weeks (participants only) and post intervention and invited to comment on their usability. All participants and carers were qualitatively interviewed before intervention delivery about their expectations and participants; nominated carers and facilitators were all interviewed after cessation about their experiences.

**Results:**

The manualised intervention and modes of delivery proved acceptable to participants and carers. Reported benefits included increased confidence and self-efficacy, engagement in new or lapsed activities and re-engagement in fun and friendships. People with dementia and carers were able to self-complete all outcome measures, but time required to complete the measures is a key factor. Strategies for recruitment need to include direct contact within 24–48 h post invitation to the study. Analysis of data on the primary outcome did not reveal any trends. Facilitators found the training and support to be appropriate and helpful.

**Conclusions:**

The tailored intervention reportedly met the needs of all participants. The study confirmed the need for careful identification and application of patient-reported outcome measures. Outcomes to measure some dimensions of reported benefit are not available.

**Trial registration:**

Current Controlled Trials ISRCTN67209155.

## Background

Dementia is a global and UK policy priority [1–3]. However, despite the policy focus and drive for early identification, the extent of unmet need amongst those who receive an early diagnosis is significant [4]*.* The impact of diagnosis and the symptomatology associated with dementia can lead to a sense of helplessness and apathy, increasing risks of depression in both people with the condition and their carers [5]*.* This together with decreased opportunities for participation can in turn result in reluctance to participate in life and associated rapid deskilling on the part of the person with dementia.

Evidence suggests that psychosocial interventions can play a significant role in supporting people post diagnosis [6] and in particular interventions that involve the person with the diagnosis and their caregiver with the aim of improving the quality of life of both [7]. However, questions remain about the extent to which speed of deterioration (which is highly specific to each person) might be ameliorated through psychosocial interventions.

Self-management is one form of psychosocial intervention, whereby those with a long term condition are encouraged to manage their physical and mental health by identifying solutions that meet their own needs, usually in partnership with professionals. Self-management programmes are well established for people with long-term conditions, but their use with people with early stage dementia is a relatively recent concept [2, 8, 9].

A preliminary study, in partnership with people with dementia and their carers, identified the potential value of a self-management intervention which would incorporate a tailored, health promoting approach to people post diagnosis. The consultation resulted in the draft ‘Journeying through Dementia’ intervention [10] which is designed to be delivered by occupational therapists.

Journeying through Dementia is based on the Lifestyle Matters programme [11] which was developed for the UK setting from a US intervention, Lifestyle Redesign. This intervention was found to promote physical and mental health and well-being, occupational functioning and life satisfaction in older adults [12, 13] as well as retaining approximately 90 % therapeutic gain up to 6 months post intervention [14].

In common with Lifestyle Redesign and Lifestyle Matters [13, 15], the intervention encourages continued participation by people following a dementia diagnosis [15] and is underpinned by social cognitive theory thereby aiming to increase participant self-efficacy [16]. Self-efficacy has been identified previously as a possible protective factor improving motivation and emotional well-being. Self-efficacy can lead to effective problem solving, followed by an increase of positive emotions and life satisfaction [17]. A holistic approach towards self-management can therefore promote self-efficacy which in turn can prevent decline [16].

The study described in this paper sought to examine the feasibility of a future population-based larger trial of this intervention. This included determining staff training and supervision requirements for delivery. The appropriateness of recruitment methods and usability of a range of potential outcome measures were also examined for application in a future trial.

## Methods

A single-site feasibility study was undertaken following the methodological framework stipulated in the Medical Research Council guidelines for development and evaluation of complex interventions [18]. According to the MRC guidelines, the feasibility stage of a trial provides essential preparatory work to test procedures for acceptability, compliance, recruitment, intervention delivery and retention. The aims and objectives of this study were designed to explore these factors in order to inform the design of a larger randomised controlled trial. In line with the guidance, a mixed method approach was also adopted to identify facilitators and barriers to participation.

### Aims and objectives

The primary aim was to explore the requirements for a future randomised controlled trial. The objectives to meet the aim were to

1. Determine the appropriateness of potential outcome measures for a future study
2. Identify the knowledge and skill set required to effectively facilitate the programme
3. Finalise the content and delivery methods for the facilitator training programme
4. Explore viability of proposed recruitment methods to a future study
5. Explore the characteristics of those who might benefit from the programme
6. Determine the optimum length of programme delivery and mode(s) of delivery
7. Further refine intervention content

**Fig. 1:**
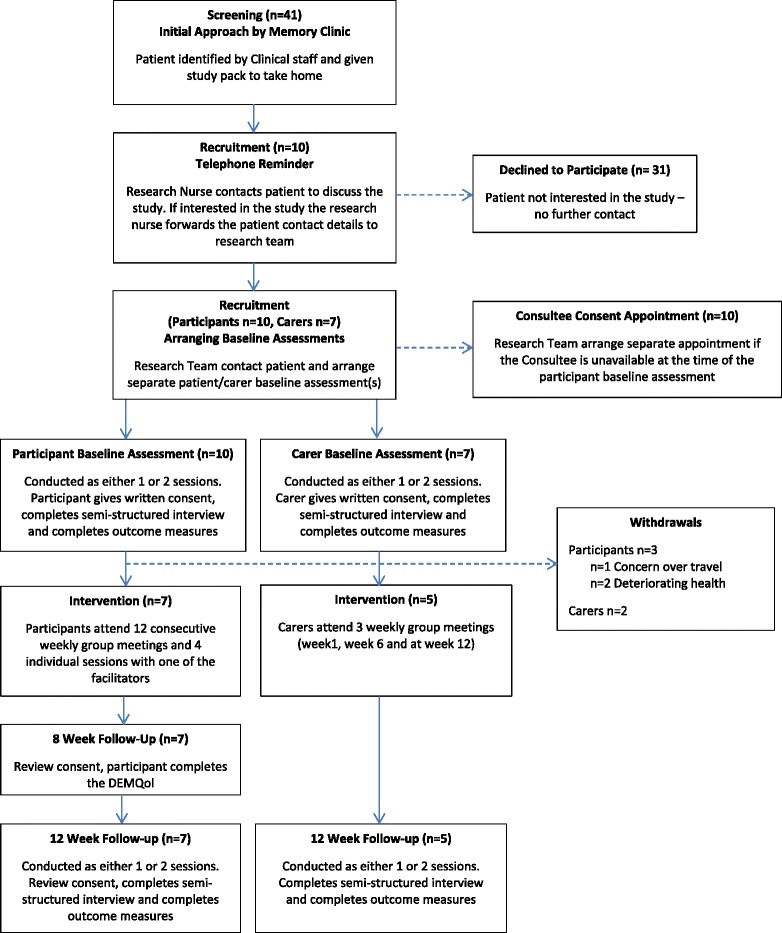
CONSORT flow diagram for the Journeying through Dementia feasibility study

A CONSORT-style flow diagram is provided in Fig. 1 [19] to illustrate the study design.

The study was approved by Yorkshire & the Humber; Leeds West Research Ethics Committee, Sheffield Health & Social Care NHS Foundation Trust and Sheffield Teaching Hospitals NHS Foundation Trust. It is registered with Current Controlled Trials, reference ISRCTN67209155 as part of a programme of work.

### Participant recruitment

**Fig. 2:**
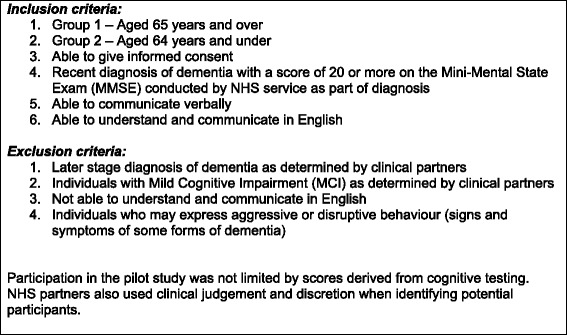
Eligibility criteria for the Journeying through Dementia feasibility study

Participants with early stage dementia were recruited post diagnosis through memory services in one city. Information about the study was given to those who met the eligibility criteria and expressed interest (see Fig. 2). Suitability for participation was assessed by the Mini-Mental State Examination (MMSE) [20] as this instrument is deemed to be appropriate for measuring cognitive capacity in larger studies as part of eligibility criteria [21, 22]. In addition, clinicians used their professional judgement when suggesting involvement. A carer information sheet was also provided.

Those who agreed to participate were then invited to involve a nominated family carer if they wished to do so, but this was not a condition of participation. Nominated carers had to be prepared to take part and also agree to complete a small number of selected outcome measures to determine their appropriateness including their usability. They were also invited to take part in pre and post intervention semistructured interviews.

### Ethics and mental capacity

Participants were also asked to nominate a consultee who would act on their behalf, should their ability to give informed consent change during the course of the study. All participants, carers and consultees provided written consent for their involvement [23, 24].

In accordance with the Mental Capacity Act 2005, assessment of capacity was reviewed throughout the study [23]. The knowledge and experiences of people with dementia and their carers were recognised and respected throughout, and participants were able to withdraw at any time.

### Intervention content and delivery

The intervention is a 12-week manualised participant-directed self-management programme. Content includes (but is not limited to) the following topics:

- Beginnings: Introductions, principles of self-management, exploring differences
- Ways of thinking about dementia: What is dementia, effects on everyday life, challenging stereotypes, sharing coping strategies
- Keeping physically well: Relationship between physical and mental well-being, embedding healthy activity in everyday life and diet
- Memory: Strategies to aid memory, impact on everyday life, learn and practise new techniques
- Keeping mentally well: Relationship between anxiety and memory and dementia and stress
- Endings: celebration of achievements and how to move forward

Delivery entails up to 12 participants (people with dementia) meeting weekly for approximately 2 hrs at a local community venue (which is assessed for suitability). Participants are facilitated to select topics of most relevance to them from the menu. Subsequent topic exploration involves a mix of didactic teaching, peer sharing and active experimentation with support from each other and from the facilitators. For example, an overall group goal could be using public transport in which case a community visit is organised together involving travel. Examples of associated activities that members identify as being valuable to pursue might include route finding and discussion of strategies for managing cognitive challenges in the community.

Participants also receive four individual sessions with one of the facilitators to pursue personal goals. The first of these individual sessions takes place before the group starts to introduce the participant to the programme and begin relationship building. The remaining three sessions take place at regular intervals over the 12 weeks. Enhanced techniques are used where necessary for communication and to scaffold memory to promote engagement [25, 26]. These include the use of cue cards, written materials providing summaries of meetings and diaries for appointments or meeting dates.

Preparatory work confirmed the importance of involving family carers. Nominated carers are therefore invited to join the group on three occasions: the first, the middle and final group meetings and engage with individual goal setting sessions if participant goals involve them.

### Facilitators and facilitation

Two occupational therapists were trained to deliver the intervention, and two student occupational therapists were recruited and trained to provide cover for absence, thereby guaranteeing delivery.

Facilitators received weekly supervision during intervention delivery, provided by experts. This was delivered in face-to-face meetings and via the telephone when necessary.

Training content was determined through the previous modelling work [10]. Facilitators and participants who took part in the preliminary study were able to provide guidance on what should be included in a training package and the mode of delivery based on their experiences. It involves a 1-day experiential format with a mix of didactic teaching and experimental group work and reflective feedback, thereby replicating the intervention [27]. The intervention is designed to be delivered by occupational therapists, but an important question for future delivery is whether other healthcare professionals can be trained and supported to deliver it. Therefore, facilitators were asked to keep reflective diaries and maintain records of their involvement in addition to participating in post intervention focus groups.

### Outcome measures

Identified measures for the person with dementia to complete were

- Dementia Quality of Life Questionnaire (DEMQoL): UK developed scale to measure quality of life for people with dementia with both self-complete and proxy completion versions [28]. A higher score (min 28–max 112) represents better health-related quality of life.
- European Quality of Life 5 Dimensions (EQ-5D-5L): widely used measure of health outcomes with utility for cost analysis [29].
- Patient Health Questionnaire-9 (PHQ-9): a widely used measure of mood [30].
- General Anxiety Disorder-7 (GAD-7): a widely used measure of anxiety [31].
- Generalized Self-efficacy measure (GSE): to assess self-belief and coping mechanisms [32].
- Physical Self-Maintenance Scale Self-rated Version (PSMS): measure of ability to perform everyday functions independently [33].
- Instrumental Activities of Daily Living (IADL): assesses independent living skills and daily functioning [33].
- A bespoke Health and Social Care Resource Use questionnaire.

Selected measures for consented carers to complete were

- EQ-5D-5L [29].
- Zarit Burden Interview [34].
- Sense of Competence Questionnaire (SSCQ) [35, 36].
- PHQ-9 [30].
- A bespoke Health and Social Care Resource Use Questionnaire.

### Study data collection methods

Consent for participation in the study was followed by a request to complete the battery of measures at baseline. This could be split across two appointments if an individual became fatigued and could take place at their home or another venue, depending upon their choice. A carer could be present at the request of the person with dementia.

If a carer was also interested in participating, the researcher arranged a separate appointment for consent and measure completion.

At each measurement time point, all participants and carers were encouraged to provide feedback to the researcher on the assessment who made written notes and also observed ability to self-complete. Of particular interests were usability, ability to understand the questions being asked and make a response, and completion tolerance to individual measures and the entire battery.

People with dementia and carers were also interviewed qualitatively at baseline about their expectations of the programme before joining and again within 2 weeks of cessation of the programme about their experiences. Schedules were developed to guide interviews and enable people to express themselves in their own terms. Topics included

- Understanding and expectations of the programme.
- Acceptability and usability of the outcome measures.
- Experience of the programme and facilitator delivery.
- Barriers and facilitators to taking part in the programme.
- Effectiveness of the programme (group and individual sessions).

### Analyses

Results from the outcome measures were examined for usability together with researcher observations. The analyses undertaken were mainly descriptive to ascertain participants and carers ability to complete the measures and the time taken to do so. Validity of scores was considered to determine their use in a future trial. The findings were analysed using the statistical package ‘R’ version i386 3.0.3.

Both baseline and post intervention interviews were audio recorded (following permission). A sample of transcripts (30 %) were read for familiarisation and annotated for themes by two researchers. Agreed themes were then used to produce an overall index for in-depth integrated analysis within and across all transcripts using Framework Analysis in NVivo [37, 38].

Facilitator experiences (including those of recruited students) were explored through a facilitated focus group held immediately post intervention. All attendees were provided with a list of topics for discussion prior to the session to stimulate and maximise feedback. Topics included skills and knowledge required to deliver the programme, quality of training and supervision, experience using the manual and barriers and facilitators to participation.

## Results

### Patient reported outcome measures

Ten participants and their carers agreed to be interviewed at baseline. Table 1 provides details of those who took part.

**Table 1:** Demographic data for Journeying through Dementia participants and carers

		Participants		Carers	
		Baseline (*n* = 10)	Follow-up (*n* = 7)^a^	Baseline (*n* = 7)	Follow-up (*n* = 5)^b^
Sex	Female	5	4	6	4
	Male	5	3	1	1
Age	40s	0	0	1	1
	50s	0	0	2	2
	60s	1	1	1	0
	70s	4	2	1	1
	80s	5	4	2	1
Ethnic group	White British	9	6	6	4
	Caribbean	1	1	1	1
Living arrangements	Spouse/partner	5	3	6	4
	Alone	5	4	1	1
Housing tenancy	Own outright	8	7	5	4
	Tenancy	2	0	2	1
Occupation	Employed/self-employed	0	0	3	2
	Retired	10	7	2	1
	Looking after home	0	0	1	1
	Other	0	0	1	1
Transport	Owns car	5	4	5	4
	Public transport	5	3	2	1

^a^Three participants withdrew prior to the group commencement

^b^Two carers withdrew prior to group commencement

The seven participants who subsequently took part in the group then completed the DemQoL at 8 weeks and the entire battery of measures post intervention.

All participant and carer scores for all outcome measures are reported in Table 2. Due to the small sample size in this feasibility study, results of the quantitative analysis were inconclusive.

**Table 2:** Participant and carer outcome measure scores by time point

Outcome measure	Stage	Completed	Mean	SD	Min	Max
Participants						
ᅟDemQol	Baseline	10	89	11	72	105
	8 weeks^a^	7	94	7	87	101
	Post intervention	7	94	12	69	106
ᅟEQ-5D-5L	Baseline	10	0.79	0.04	0.72	0.85
	Post intervention	7	0.77	0.09	0.65	0.88
ᅟPHQ-9	Baseline	10	6	6	0	19
	Post intervention	7	3	3	0	9
ᅟGAD-7	Baseline	10	4	5	0	18
	Post intervention	7	1	1	0	4
ᅟGSE	Baseline	10	27	5	20	36
	Post intervention	7	25	9	10	34
ᅟPSMS	Baseline	10	23	0	23	23
	Post intervention	7	23	0	23	24
ᅟIADL	Baseline	10	6	2	2	8
	Post intervention	7	6	2	3	8
Carer						
ᅟEQ-5D-5L	Baseline	7	0.81	0.30	0.16	1.00
	Post intervention	5	0.75	0.38	0.09	1.00
ᅟPHQ-9	Baseline	7	5	6	0	15
	Post intervention	5	7	10	0	23
ᅟZarit Burden	Baseline	7	16	13	2	40
	Post intervention	5	24	18	4	52
ᅟSCQ	Baseline	7	31	6	19	35
	Post intervention	5	26	8	17	34

^a^The primary outcome, the DemQol, was also repeated at 8-week follow-up

**Fig. 3:**
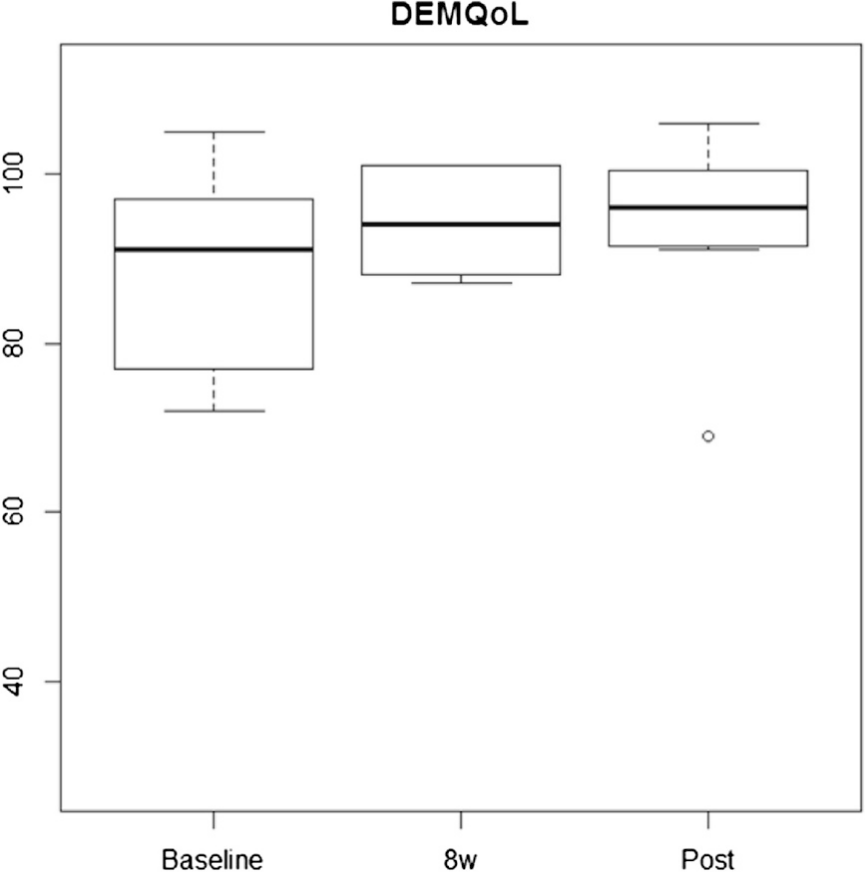
Participant DEMQoL distribution of scores at baseline, 8 weeks and post intervention

Figure 3 illustrates the distribution of scores for the primary outcome, the DEMQoL, at baseline, 8-week follow-up and post intervention. Although small, the mean score was found to increase from baseline to 8-week follow-up and this was maintained post intervention. The greatest change appears to be for those participants who scored lower on the DemQol at baseline.

### Facilitator training and programme facilitation

Training and supervision reportedly equipped these experienced facilitators with the skills to deliver the programme, although less qualified staff may require specific training on dementia. Weekly supervision was considered important for guiding facilitators on tailoring the programme and planning sessions, and the level of support required was dependent on the experience of the facilitator. A higher level of supervision was preferred at the start of delivery when more guidance was needed. Supervision was considered most effective when undertaken directly after group delivery when issues were current.

Style of facilitation was very important to the participants, who described the facilitators as personally engaging,

They were fun and they were, you felt confident in them. They were imaginative. they made things fun and I think that was a big part of it (Participant).

Good quality facilitation was also important for encouraging attendance,

I thought it was run exceeding well, in fact if it hadn’t done I might have made an excuse and not go. But no I was always ready to go (Participant).

Post-intervention delivery, facilitators advocated a ‘client centred’ (Facilitator 1) approach in congruence with the underlying ethos of the programme.

### Recruitment methods

Two groups were originally planned for the feasibility study, one for those aged 65 years and over and a second for participants aged 64 years and under. A total of 20–24 participants were therefore sought, 10–12 per group. Recruitment of those 64 years and under proved unsuccessful due to a limited number of participants being identified and recruited within the necessary time frame.

Initial low recruitment rates overall identified the need for direct phone contact within 24–48 h following receipt of information to ensure that the potential participant had read and understood what their involvement would entail.

Ten participants aged 65 years or over were successfully recruited and completed baseline assessment. Three then withdrew from the intervention prior to the group starting.

Branded study materials appeared to be an effective memory aid for participant recall during assessments. Information was deemed adequate to make an informed decision to take part and the time and opportunity to discuss the study with a researcher on a one-to-one basis was appreciated,

You being here asking the questions has made it slightly easier for me to make me mind up (Participant).

Time and further explanation were required for the legal and ethics sections of the information sheet.

### Who might benefit from the programme?

One group was delivered to seven people with dementia with five also having a family carer involved. The participation of carers was not related to any particular participant characteristics, for example, those with worse symptomology.

Attendance rates across the 12-week programme were 87 %. Reasons for non-attendance were either illness or attending hospital appointments. The minimum number of weekly meetings attended was nine and the maximum was 12. Six of the seven people with dementia took part in all four individual sessions. The seventh attended three and missed the fourth due to a fall. Nearly all participants were transported to and from the group by a relative or used public transport. Several participants were car owners and able to drive.

Group member diversity was described positively by participants,

They were a lovely group of people to meet with as well. Totally different from each other (Participant).

Having people from different backgrounds and lifestyles with a range of dementia severity was even thought to improve group interaction by carers,

They were different people from different groups, from different lifestyles. I think that everybody was able to give something… (Carer).

The group was considered to be a safe place to meet with similar people without prejudice, for exploring new ideas and experimenting with activities*,*

a level playing field with this thing (Participant).

Although the group generally worked well together, participants did describe how the impact of dementia affected the contribution some participants were able to make to the group. This was noticed during involvement in activities as well as contributing verbally to discussions. However, this does not appear to have been detrimental to group bonding,

We’ve said in fact again today, in fact nearly every week we have said we have been lucky because there’s only [name] who hasn’t been able to mix with us. You know, she’s sat and listened, she’s ever so sweet bless her but she just doesn’t…didn’t talk at all. If you spoke to her she’d answer you but you couldn’t get her to talk er but the others we’ve all chatted together (Participant).

### Optimum programme format and delivery

Facilitators considered the length of the intervention, 12 weeks, to be adequate to equip participants with the tools required to maintain learned skills without developing dependency,

If it goes on for too long people get too needy of you and that expectation, the longer it goes on the harder it is for them to that see you not being there (Facilitator).

Although the participants talked about continuing to meet up following the end of the programme, the majority expressed some disappointment that the programme could not be permanent or at least continue in a different format,

It would be nice to have a session…not frequently but probably every what 3 2 or 3 weeks? (Participant).

The size of the group supported inclusive engagement through sharing and listening,

I think it was just right. I think had there been a lot…it wouldn’t have been as intimate and had it been less we might have felt a bit less able to talk so I think as it was everybody got a chance to speak if they wanted to… (Participant).

A valued aspect of the intervention was the warm welcoming environment and selection of a suitable venue supported this ambience,

It had a feeling about it where this type of meeting belonged…The environment was most important (Carer).

I think it was ideal. With a great big table in the middle and a cup of coffee if we wanted it all there beside us. No I think as a venue it was brilliant (Participant).

Inappropriate venues, in particular busy, noisy places, could have a significant impact on the delivery of the intervention,

We didn’t like it when we got moved somewhere else…Especially to the University café because there’s so much going on and coming and going and bits happening (Participant).

The chosen venue was centrally located with access to parking and public transport links which alleviated concerns about getting to the group. Facilitators also agreed using a local venue could support long-term transition of the group to self-management after programme cessation.

Participants described how the inclusion of outings, rather than just venue-based activities, provided opportunities to explore the wider community, put experiences into practice and build confidence. This particular group visited their local art gallery and city library,

… we all realised that we were still capable of…going out and about er which is something I must admit I had stopped doing… (Participant).

Prior to involvement, all ten people with dementia described being keen to remain independent and saw the programme as an opportunity to support this goal. This aim was supported by the carers. Poor memory was cited by both participants and carers as being the main cause of loss of confidence and increased anxiety. Pre-programme pre-conceptions of memory capacity were therefore felt to be an indicator of ability to engage in the programme and a consideration for how beneficial it would be,

Yeah I think it would depend on…how much he would take on board and retain…how useful it would be for him (Carer).

Post intervention participants were surprised at their ability to engage in and implement the programme content either by themselves or with the support from their family,

They made us realise, you know, it wasn’t the end of the world, that there was still life to live and I think some of us, probably myself included, had just thought, that’s it. End of life, nice life doing things. I think it did give people a real feeling that, oh there was hope for the future. There were things that we could still do, there were places we could still go to and you could meet together (Participant).

The planned involvement of carers was described as enabling facilitators to establish clear boundaries for carer involvement, allay any anxieties and provide carers with the information they required to support the person they care for in taking forward learning out of the programme.

### Intervention content

Compared to other groups, participants did not feel patronised by the intervention*.* The flexibility of the intervention through the manualised programme was enjoyed by the participants. In particular, how one topic could lead to different, unplanned topics and that the format of the programme allowed this diversity,

We did some and we’d go off a bit and come back to it sort of thing which is…it’s not like being at work or being at school where that’s got to be done… (Participant).

Individual sessions were valued by facilitators as an opportunity to observe family dynamics, identify individual needs and discuss expectations of the programme. Goal setting during these sessions was found by the facilitators to be a difficult concept for participants to understand; however, using examples and discussing ‘achievements’ rather than goals meant, participants were able to explore and articulate their ideas. Goals identified by participants included cooking, reading and memory techniques such as using a daily diary and using a bookmark on which to write a brief synopsis of storylines and characters as a memory aid.

Carers’ sessions were well attended, and participants considered that these were important for engaging carers,

…because it keeps them more in touch with us… (Participant).

However, several carers wanted more detailed information on implementation of activities,

…so if the papers that they sent or came home with were a little bit more explicit instead of just saying we talked about [topic] to actually say what they were actually doing about it (Carer).

Tools and techniques, for example daily diaries, memory cards and re-usable activity cards, explored as part of the programme appeared to increase self-efficacy,

Every week I think you learnt something new and so it made it worthwhile going (Participant).

Participants explicitly described how the programme influenced a change in attitude from dwelling over the past to looking to the future,

…I do feel that I’m looking forward to things more, no matter what they are, I’m looking forward to them er so I suppose it’s, going there has helped me to erm get a better grip of myself'…I’m not walking about with a dull expression on my face or a dull thinking. I’m thinking more positive (Participant).

Having fun and laughter as part of the group were also important to participants as a way to engage with the programme and with other group members thereby de-stigmatising dementia,

We would all laugh together or sympathise together and I think that was a very valuable part of the group because if someone hasn’t experienced even anyone in the family having Dementia they’ve got no idea. Inside you’re the same person” and “I mean listening to us talking you wouldn’t have known that there was anything different about any of us (Participant).

Continuation of the group after programme cessation was cited as very important due to a perceived lack of available similar groups,

… it’d be nice to meet up because there’s nobody…there’s no group round here like it…I think they’ll probably find a vacuum now that it’s finished and it’d be nice to have…somewhere to, once every so often, to go and meet up with them (Participant).

However, establishing deeper bonds was considered essential for continuation and could not be reliant on a shared diagnosis of dementia alone,

It will be very interesting to see how long this group just meeting on their, you know, on their own with only that motivation that you, all, you share together your problems with memory loss, erm how long it will go on… (Participant).

It was clear that many of the carers felt that for the group to continue a ‘leader’ would be required,

I think that’s good [group continuation] as long as they’ve got somebody there that can take the lead (Carer).

## Discussion

### Participant reported outcome measures

Findings from both the quantitative and qualitative analysis for participants and carers demonstrated feasibility of use and ability to complete a range of outcome measures about general health and well-being. The overall battery was not found to be burdensome, and all people with dementia were able to self-complete all selected measures. However, dementia severity did affect time required to complete the assessments. Completion of factual questions e.g. such as medications they are taking for the Health and Social Care Resource Use questionnaire was problematic if a family carer was not present. Carer presence also appeared to reduce anxiety but could also potentially lead to bias, influencing the openness of responses. Several dyads disagreed over questionnaire responses. Overall tolerance to a variety of outcome measures designed for use with people with dementia was good and supports their future use in an RCT.

The primary outcome, the DemQol, demonstrated an improvement in the mean score at 8 weeks and showed a very small decline post intervention. The findings were reflected in the qualitative data in which participants and carers described enjoying the intervention, followed by disappointment and sadness when it ended.

Currently, there is a lack of standardised specific instruments for measuring positive concepts in dementia [39] and reliable measures for capturing change in social support and social networks. However, consideration should be given to inclusion of such measures in a larger RCT. A potential measure which has demonstrated good validity would be the Lubben Social Network Scale (LSNS-6) which has been used with community dwelling older adults [40] and people with early stage dementia [41].

The type of respondent, including good orientation, attention and language skills, has been cited as more important than cognitive ability in self-complete dementia quality of life instruments [22, 42]. Reproducibility has been shown in studies with larger participant numbers [43]. What is important is the inclusion of participants in outcome completion, rather than relying on proxy completion, due to the subjective nature of these measures [44].

A recent diagnosis of dementia means at this stage people are coming to terms with the diagnosis. Researchers with experience of working with people with dementia must have the knowledge and skills required to ensure outcome measures are recorded as consistently as possible. This should include assessment of the reliability of participant responses [22]. Alternatively, good quality training is essential to develop these skills if inexperienced assessors are involved.

### Facilitator training and programme facilitation

The 1-day training programme and weekly supervision were found to be effective for occupational therapists with prior experience working with people with dementia, enabling them to feel sufficiently supported to deliver the intervention. Further training on dementia as a condition and provision of good quality supervision may be required for less qualified staff. In particular, supervision is likely to be required to manage the challenges of allowing participants with dementia to take control of the programme and make personal decisions about risk taking [10]. Interestingly, more important than professional qualifications were the personal characteristics of the facilitators and their delivery style which were found to be a motivator in participant attendance. Getting the right facilitators in place is therefore a key to successful delivery. Implications for a future study would be to identify staff with the necessary professional skills as well as personal skills to deliver the programme.

### Recruitment methods

Recruitment to this study raised a number of key issues. Firstly, although there was interest from people aged 64 years and under, the overall numbers in this category were too small to be feasible for a group intervention specifically for this age group. A group for people aged 65 and over was viable and accords with the greater prevalence in people aged 70 years and over as reported by Alzheimer’s Research UK [45]. Age limits did not appear to be necessary and therefore any future trial would not set a defined age range for inclusion. Secondly, identification of potential participants was conducted solely through memory services, with no other strategies employed such as through mental health charities which could be considered for future research. However, alternative methods of recruitment would need consideration in order to capture individuals with early stage dementia. Thirdly, the information pack provided by clinicians to participants was found to be fit for purpose. However, in order to enhance recruitment, direct follow-up in the form of a telephone contact is required to help remind potential participants to read the information pack. Without this contact during recruitment, a number of participants may not have taken part. Fourthly, a balance is required to ensure carers do not feel excluded, but not allowing carers to become a focus of the intervention [10].

### Who might benefit from the programme?

Adults with early stage dementia were demonstrated to successfully engage in a person-centred self-management group intervention. Journeying through Dementia appeared to meet the heterogeneity of needs of people with dementia by being tailored to their individual requirements as well as those of the overall group. This was evident during group meetings where content was selected by the group and discussions guided by what was identified as being important. Diversity amongst group members including age, lifestyle, background and understanding of dementia was considered a benefit and contributed to the enjoyment and learning experience of the programme. Capacity to participate in the intervention was considered to be influenced by memory. The benefits of taking part in the Journeying through Dementia intervention may therefore be affected by how well participants were able to engage with the recruitment and intervention materials.

Participant ability to engage in the intervention and contribution to the group did appear to be influenced by their dementia. What became clear was that the support from a third party, in this intervention the carer, was required to support participant continued engagement in learned activities outside of the group and potentially for continuation of the group. Therefore no amendments will be made to intervention design for a future study, but will continue to explore the level of engagement from carers and facilitators.

### Optimum programme delivery

The manualised programme was implemented by the facilitators, and participants were able to select and explore topics of interest to the group. Individual sessions identified a range of valued activities with participants and pursued outcomes to enable re-engagement. Activities maintaining independence and hobbies were identified by this particular group including cooking, remembering appointments and reading. A number of participants also shared their ideas and successes from the individual sessions with the wider group in order to benefit everyone.

Although the length of the intervention was considered appropriate to impart skills to participants, it is essential that a dependency on the facilitators to lead the intervention does not develop. Group size should be no more than 12 to ensure facilitators have enough time and resources to support the group and deliver individual sessions during the intervention. However, too few and the sharing and learning experience of the intervention could suffer.

The original UK Lifestyle Matters intervention was 16 weeks in duration. Based on current NHS provision of group programmes for dementia patients, the intervention was reduced in-line with current service provision. The purpose was also to test whether the intervention could be successfully delivered in 12 weeks to people with early stage dementia, with an interim test at 8 weeks to explore whether the duration could be reduced even further.

It is anticipated that people with a recent diagnosis may need more support at the start of their treatment and therefore may go on to develop dependency on the facilitators and the intervention. The concept of ‘excess disability’ in dementia suggests that the level of perceived incapacity experienced by an individual, or dysfunctional treatment of that individual by others, can lead to greater actual impairment in his or her abilities [46]. Once a diagnosis has been confirmed, the relationship between the person with dementia and a caregiver can alter, leading to both character and attitude change [47]. As a self-management programme, the theory suggests that as participants develop greater confidence and self-efficacy, this will actually reduce dependency on the facilitators and the group and instead the group becomes another support mechanism along with family, the community and care providers [9]. As demonstrated in the results section, participants expressed enjoyment of the intervention and a wish for it to continue, which they facilitated themselves post intervention. This suggests that the intervention could be successfully delivered in 12 weeks with this particular group and that they harnessed their own and the group’s resources to continue without dependency on facilitator support.

The qualitative evidence indicated that a number of benefits experienced by participants included increased self-confidence and self-efficacy post diagnosis [16]. Opportunities to go on outings, have thoughtful discussion, share ideas or concerns and develop new friendships were also identified by participants as positive outcomes of the programme. Supporting this assumption was group continuation after intervention cessation. The group met on at least two occasions post intervention at a local café. These meetings were facilitated by one member of the group. Although other programmes are available to participants via memory clinics and charitable organisations, the Journeying through Dementia intervention was thought to provide a novel option not currently available in the community. Participants felt the intervention was more about the person than about the condition and how they were still a functioning member of society and could retain life skills regardless of their dementia.

Participants and carers agreed that the carer attended sessions were informative and prevented feelings of exclusion. Engagement was also required if carers are to support the participant in further developing and sustaining skills after the programme ends. Facilitators indicated that although a person-centred programme, it was important to get carer buy-in which was provided by the carer sessions [10]. Participant and carer’s trust in the facilitators was found to be an essential factor in programme attendance and engagement.

### Content

Facilitators engaged with the manual and felt able to adapt suggested topics with the group to meet their needs. During this study, no new topics were identified by participants for the manualised programme. Further topics may be identified as part of a larger study with more participant input.

Facilitator, participant and carer’s understanding of the purpose of the intervention is a key to its delivery. Terminology may need some revision, for example, an alternative to use of the term ‘goal setting’, to better engage participants. This could be managed on a group by group or individual basis.

Participants described the intervention as a fun and safe environment in which people with early stage dementia could meet, share and learn. The foundation of which supported learned activities post intervention and continuation of the group, who had already met and had further sessions arranged after intervention cessation.

## Conclusions

The purpose of this study was to ascertain the feasibility of conducting a larger RCT in the UK setting, and this was achieved. In addition, to further exploring the benefits of the intervention on a larger sample size, a future trial should also conduct a health economic evaluation to establish the cost-effectiveness of such an intervention and also include a measure to capture change in social support and networks.
